# The Cambridge Discussion Summarised

**Published:** 1920-07-10

**Authors:** 


### THE FUTURE OF MEDICAL PRACTICE.

### The Cambridge Discussion Summarised.

Decidedly the most important discussion from the point of view of State medicine, at the Annual Representative Meeting of the British Medical Association at Cambridge, took place in the Section of Medical Sociology, which was presided over by Dr. George E. Haslip. Here a discussion was opened on the future of medical practice. Sir George Newman discussed the claims of the State upon the doctor, and also had something useful to say on the future of the hospital system. He said that, no doubt, from the medical point of view, the community stood in urgent need of more hospital accommodation. But the solution was not as simple as it looked, and the situation was complicated by many other hospital problems too often neglected. Was the principle of voluntary support to continue in our hospital system, or was the medical profession in favour of Government aid and some measure of Government control? Ought the hospital accommodation of the community to be allocated by the State in respect of area or type of clinical work undertaken, or ought it to continue to be provided in haphazard fashion? Was the medical and surgical staff to remain honorary? Was it to be a special professional class, or composed, in part at least, of general practitioners? What was to be the relation of hospitals to each other, and to contributory clinics, and to the insurance system? How were we to bring about the substantial reform of the out-patient departments which was so greatly needed? Ought we to make provision for paying patients, and, if so, on what basis? Could we fairly provide for the insured and the pauper whilst neglecting to provide for the remaining majority? And, lastly, what could be done to re-organise after-care and the proper convalescent treatment of the patient? Sir George Newman contented himself with stating the problems without attempting to solve them. For the right answer, he said, medical as well as lay judgment and experience were required.

### The Consultant's Point of View.

Sir Wilmot Herringham followed with a paper from the point of view of the consultant. The whole of his paper should be studied by those interested in the hospital question. At the present time, he said, there were two classes who obtained the best treatment that was to be given in the country. They were the very rich and the very poor. But the middle class came off very badly. The only sound remedy for this and for other imperfections which he instanced was to extend to private patients the benefit and convenience of treatment at a hospital. He would unhesitatingly adopt the plan of a differentiated scale of fees, whereby the rich would be made to pay more and the poor less. A part of the general hospital should be the paying block, where the patients would be treated only by the staff. They should be entitled to use the treatment provided for the hospital, and the pathological work should be done in the hospital laboratories. But he would go further. He would provide offices for his clinical staff in which they should conduct all their private practice. They should come down in the morning to their work just as a barrister went to his chambers, and their whole business day should be spent within the place.

After Dr. Alfred Linnell had spoken from the standpoint of the general practitioner, and Professor Gowland Hopkins from that of medical research, Mr. E. W. Morris, C.B.E., spoke on the hospitals, with Lord Knutsford as an appreciative listener. Mr. Morris's contribution was in many respects the most telling of the morning, and an abstract of his remarks will be found on another page (p. 374). He gave an account of the experimental units of this kind which had been inaugurated at the London Hospital. He was no believer in what was called the parallel system, whereby a voluntary staff was run side by side with a paid. And he believed that the time was coming when all the beds at our teaching hospitals would be under the charge of paid clinicians, and he thought this would be better for the patients, better for the students, and ultimately better for the public.

### What the Labour Party Thinks.

The section next listened to Mr. D. T. Jenkins, secretary to the Labour Party Health Committee, who pleaded for a whole-time State medical service. The Labour Party, he said, did not want the workers to be spoon-fed. The workers must be educated in precautionary and hygienic measures. Nor did his Party expect the medical profession to accept the proposal of a whole-time State medical service without being given a measure of interest in it and of control over it. It had been stated that morning that the very poor could always obtain the best treatment in the hospitals. But there was not sufficient accommodation, even with the Poor-Law infirmary thrown in. The existing hospitals must be multiplied by six before it could be said that the accommodation was sufficient. How was that to be done? Hardly by voluntary effort. There were only two other alternatives. One was to have paying patients, but that shelved the poor. The other was State aid, and he was anxious that the State should not only come to the aid of the existing hospitals, but should provide sufficient hospital accommodation for all needs in all parts of the country. If the voluntary system of hospitals was to continue, then he asked that those who were responsible for introducing patients into hospitals should not introduce millionaires into free beds. That was being done. (Lord Knutsford: No.) Mr. Jenkins went on to say that there was only one remedy. It was to have the whole nation insured and the whole nation bearing the cost.

Dr. Adam Fulton, of Nottingham, spoke of industrial practice, and of what the State could do to help the general practitioner engaged in such work, and another speaker on the same lines was Dr. F. J. Blackley, of Warminster. An account of the St. Andrew's Institute established by Sir James Mackenzie was forthcoming from Dr. Girdwood. Three panel practitioners were on the clinical staff of this Institute and used its rooms as its panel consulting-rooms, and had also the use of the special departments for their panel cases. The general practitioners each devoted two hours a day to research work, and all the research cases were discussed by the entire staff once a week. The purpose of the Institute was to conduct research into the early stages of disease. Dr. Roy Scott, of Plymouth, said that unless the general practitioner was treated with complete candour and generosity, there would be a widespread and disastrous revolt. The more the State paid the more the State would control, and the State ultimately meant the taxpayer and the ratepayer, and those who knew the

**The Future of Medical Practice**—(*continued*).

*personnel* of insurance and other local committees well understood what that would mean. Sir Kenneth Goadby dwelt upon the need for close association between the clinician in charge of the case and the laboratory worker. The general practitioner required a handy laboratory where he could not only get one examination, but could go and put a supplementary question, which might yield a more illuminating answer than the original.

The Family Doctor and Preventive Work.

Mr. E. B. Turner contended strongly that the family doctor had done a vast amount of preventive work, simply because, treating the same family year after year, he was able to detect the beginnings of disease.

Finally came Dr. Peter Macdonald, of York, who, while declaring himself enthusiastically in sympathy with the assumption by the State of the responsibility for the medical services, said that if this was done at all it must be done well and efficiently. He had tried to work out the cost for his own city of York of a complete and adequate service. The hospital service in York now cost over £13,000 a year, and he was quite clear that it would be necessary to multiply hospital accommodation, if not by six, as Mr. Jenkins suggested, at any rate by three. This would make the cost of hospital services in York alone £40,000, and that was based upon the continuance of a gratuitous medical service. The cost to the whole community of an efficient medical service would be not less than forty millions.

Sir Wilmot Herringham, in replying on the discussion, said he did not think there was any need to be anxious about the vastness of the sum required to ensure an efficient medical service for the community. It was quite evident that the Labour Party was willing to provide it, and that generally meant that it would come out of other people's pockets, so there was nothing more to be said!

